# Microsecond molecular dynamics of methane–carbon dioxide swapping in pure and saline water environment

**DOI:** 10.1038/s41598-022-06583-z

**Published:** 2022-02-16

**Authors:** Avinash V. Palodkar, Harshal J. Dongre, Niraj Thakre, Amiya K. Jana

**Affiliations:** 1 Energy and Process Engineering Laboratory, Department of Chemical Engineering, Indian Institute of Technology, Kharagpur, 721 302 India; 2 Upstream and Wax Rheology Division, Council of Scientific and Industrial Research-Indian Institute of Petroleum, Dehradun, 248 005 India

**Keywords:** Environmental chemistry, Carbon capture and storage, Fossil fuels

## Abstract

This work aims at proposing the nondestructive methane-carbon dioxide (CH_4_–CO_2_) replacement mechanism as an ecofriendly energy production technique from the natural gas hydrate reserves in seafloor and permanently frozen grounds. Although the experimental data is widely available in literature, this replacement mechanism has not been elucidated at molecular level. In this contribution, we perform the microsecond level molecular dynamic simulations to evaluate two different CH_4_–CO_2_ replacement mechanisms: (i) direct CH_4_ displacement from hydrate structure, and (ii) dissociation of existing methane hydrate followed by a reformation of mixed CH_4_–CO_2_ hydrates. For this, we analyze CH_4_–CO_2_ replacement in three different modes i.e., CO_2_ as a replacing agent in (i) absence of free water molecules, (ii) presence of free water molecules, and (iii) presence of salt ions and free water molecules. Despite slow kinetics in the first mode, pure CO_2_ is observed to replace the methane more efficiently, while in the second mode, CO_2_ forms a new mixed hydrate layer on the existing seed crystal. However, in the third mode, salt ions help in destabilizing the methane hydrate and allow CO_2_ to form the hydrates. This proves that salt ions are favorable for CH_4_–CO_2_ replacement.

## Introduction

Clathrate hydrates are non-stoichiometric ice like crystalline compounds formulated from the saturated aqueous solution of a suitable guest gas at a reasonably low temperature and high pressure. The clathrate hydrates, also known as natural gas hydrates (NGH), exist in the deep marine sediments and permafrost regions^1^. They are regarded as a potential source of tremendous cleaner energy as concentrated deposits of natural gas^2^. As of now, there are two principally different mining mechanisms (i.e., direct decomposition and replacement reaction) employed to recover the natural gas from the hydrate reservoirs^3^. In the direct decomposition mechanism, the natural gas hydrates are destabilized by (1) increasing temperature (i.e., thermal stimulation), (2) reducing pressure (i.e., depressurization) and (3) introducing the destabilizing compound (i.e., inhibitor injection). However, as NGH are responsible for maintaining the stratum of the seafloor, the use of direct-destructive methods can invite geological disasters such as earthquakes, submarine landslides, etc. These potential disasters can be overcome by employing a non-destructive mining mechanism for natural gas recovery from their hydrate reservoirs^4^.

The CH_4_–CO_2_ replacement reaction is a feasible technique for recovering the natural gas from the NGH reservoirs without affecting the stratum’s stability^5,6^. This method utilizes a potential greenhouse gas (i.e., carbon dioxide) to replace the natural gas (majorly methane) entrapped in the hydrate reservoirs. More importantly, it has already been proved that CH_4_–CO_2_ mixed hydrates are relatively stable than natural gas hydrates^7^. The feasibility of the replacement reaction is confirmed through the experimental observations and theoretical models reported in the literature^8^. In this regard, Ohgaki et al.^9^ have conducted a preliminary feasibility study and evaluated the distribution coefficients of CH_4_ and CO_2_ between the gas and hydrate phases. Here, they have observed that the CO_2_ tends to distribute more quickly than the CH_4_ in the hydrate phase. This ease is attributed to the relative size difference of CH_4_ and CO_2_ molecules with respect to the hydrate cavities. Further, by using the Raman spectroscopic analysis, Uchida et al.^10^ have observed that the interfacial surfaces between CO_2_ gas and CH_4_ hydrate crystals act as a reaction surface area. Afterward, Uchida et al.^11^ have conducted sizable formation and dissociation experiments on CH_4_–CO_2_ mixed hydrates, and it is confirmed that the equilibrium pressure required to form the CH_4_ hydrate is considerably higher than the CO_2_ hydrate at the same temperature (usually, below 283 K). This is further supported by the phase equilibrium studies conducted by Anderson et al.^12^. Another basis that supports the feasibility of replacement reaction is the fact that the formation of CO_2_ hydrates (− 57.98 kJ mol^−1^) can simultaneously supply heat to decompose the existing CH_4_ hydrates (+ 54.49 kJ mol^−1^) while preventing any further reformation of CH_4_ hydrates^13^.

On confirming the replaceability of CH_4_ with CO_2_ in the NGH, it is crucial to understand and evaluate this substitution mechanism. In this light, Yoon et al.^14^ performed in situ Raman spectroscopy on the coexisting hydrate and bulk water phase of mixed CH_4_–CO_2_ system/gases/formers. They^14^ have observed a surface shielding effect caused by the CO_2_ hydrates formed by dissociating CH_4_ hydrates on the outer layer. Therein, the same layer of CO_2_ hydrates obstructs the movement of CO_2_ gas towards the CH_4_ hydrates that are present near to core center. Such obstruction effect is directly responsible for the decrease in the replacement efficiency. Further, Ota et al.^15^ have conducted a replacement reaction in a high pressure optical cell by utilizing a saturated liquid CO_2_ as a replacing agent. Here, on employing an in situ Raman spectroscopy, it is observed that the large cages of CH_4_ hydrates dissociate relatively faster than the small cages. This affinity of CH_4_ towards the smaller cages is attributed to either its higher stability or its favorable tendency towards the reformation followed by hydrate dissociation. Later, Zhou et al.^16^ have compared the CH_4_ replacement rate between the emulsified and the liquidated CO_2_. Herein, they^16^ have noted a significant enhancement in the replacement efficiency using CO_2_ emulsion. This is ascribed to the physical and thermodynamic properties of CO_2_ emulsion, which advantageously enhance the heat and mass transport inside the existing CH_4_ hydrates.

Although the aforementioned CH_4_–CO_2_ replacement studies provide factors contributing to its efficiency, they lack to present the mechanisms of dynamics involved. Molecular dynamics simulations are used to study nucleation, stability and unusual self-preservation effects of a pure component gas hydrate in relatively distinct environments^17,18^. This system-oriented scope thus restricts these studies to the hydrate inception, its growth and collapse. However, to understand the CH_4_–CO_2_ replacement dynamics, the hydrate formation and decomposition ensemble must be specifically targeted. In this view, Qi et al.^19^ have simulated the replacement of CH_4_ from a 4 × 4 × 4 replica of unit hydrate lattice with gaseous CO_2_. This molecular dynamic study perceives the necessary dissociation of CH_4_ hydrate structure to facilitate the transfer of gaseous CO_2_ to the lattice cages. Besides, the replacement of CH_4_ molecules by CO_2_ molecules in the hydrate phase takes a relatively long time. Overall, it is proposed that the replacement process is divided into three stages i.e., (1) breaking of the already existing CH_4_ hydrates, (2) exiting or transferring of CH_4_ from the hydrate to gas phase, and (3) assembling of CO_2_ molecules with water molecules to form hydrate structure. Similar observations are reported by Bai et al.^19^ during their microsecond molecular dynamics simulation to comprehend the replacement of CH_4_ hydrate with CO_2_. Additionally, they^20^ have confirmed the existence and decisive role of the memory effect of water molecules (obtained after CH_4_ hydrate dissociation) towards the facilitation of CO_2_ hydrate formation and growth. Besides, in the view of shale gas exploitation and carbon dioxide storage, Kadoura et al.^21^ report that the preferential adsorption of CO_2_ molecules on the porous material (clay) surface as compared to CH_4_. Thus, it can favor the growth of CO_2_ hydrates during the CH_4_–CO_2_ swapping process. Recently, Matsui et al.^22^ have observed that the CO_2_ mixed with N_2_ is deeply penetrating into existing CH_4_ hydrates during the replacement process. This is attributed to the easiness of methane decomposition in presence of nitrogen and consequent improvement in an effective area for the replacement process. Besides, they confirm the preference of CO_2_ and N_2_ in occupying large and small cages, respectively, and consequently an improvement in the overall replacement efficiency with respect to pure CO_2_ case.

By attempting to analyze and present the mechanisms for CH_4_–CO_2_ replacement in hydrates, the existing studies reinforce the ‘dissociation followed by a reformation of mixed hydrate’ theory. However, the literature suggests another replacement mechanism based on solid state conversion, where either pure hydrates of CO_2_ or mixed CH_4_–CO_2_ hydrates are obtained. This is a prolonged process, which dominates only in the absence of freely available water^6,23,24^. Despite the potential candidate of high replacement efficiency, this class of replacement mechanism has never been investigated at the molecular level. Besides, there is no fundamental understanding of the distinctive nature of the aforementioned replacement mechanisms. Therefore, we attempt to comprehensively investigate and compare these two elementary classes of replacement mechanisms by performing dynamic simulations at the molecular level.

In this contribution, we investigate the replacement mechanism in configurations of (i) direct displacement of CH_4_ from its hydrates with CO_2_, and (ii) dissociation followed by the formation of mixed CH_4_–CO_2_ hydrates. To represent the first mechanism, we conduct dynamic simulations in absence of free water molecules. We then introduce free water molecules in the simulation box with CO_2_ to investigate the ‘dissociation of CH_4_ hydrate followed by the formation of CH_4_–CO_2_ hydrate’ type of replacement mechanism. To investigate the effect of seawater, salt ions are introduced in the free water system. Finally, we evaluate these replacement dynamics in the view of reservoir operating pressure of 10 MPa. The conducted microsecond dynamic simulations scrutinize and compare the fundamental behavior of replacement mechanisms under diverse configurations.

## Results and discussion

**Figure 1:**
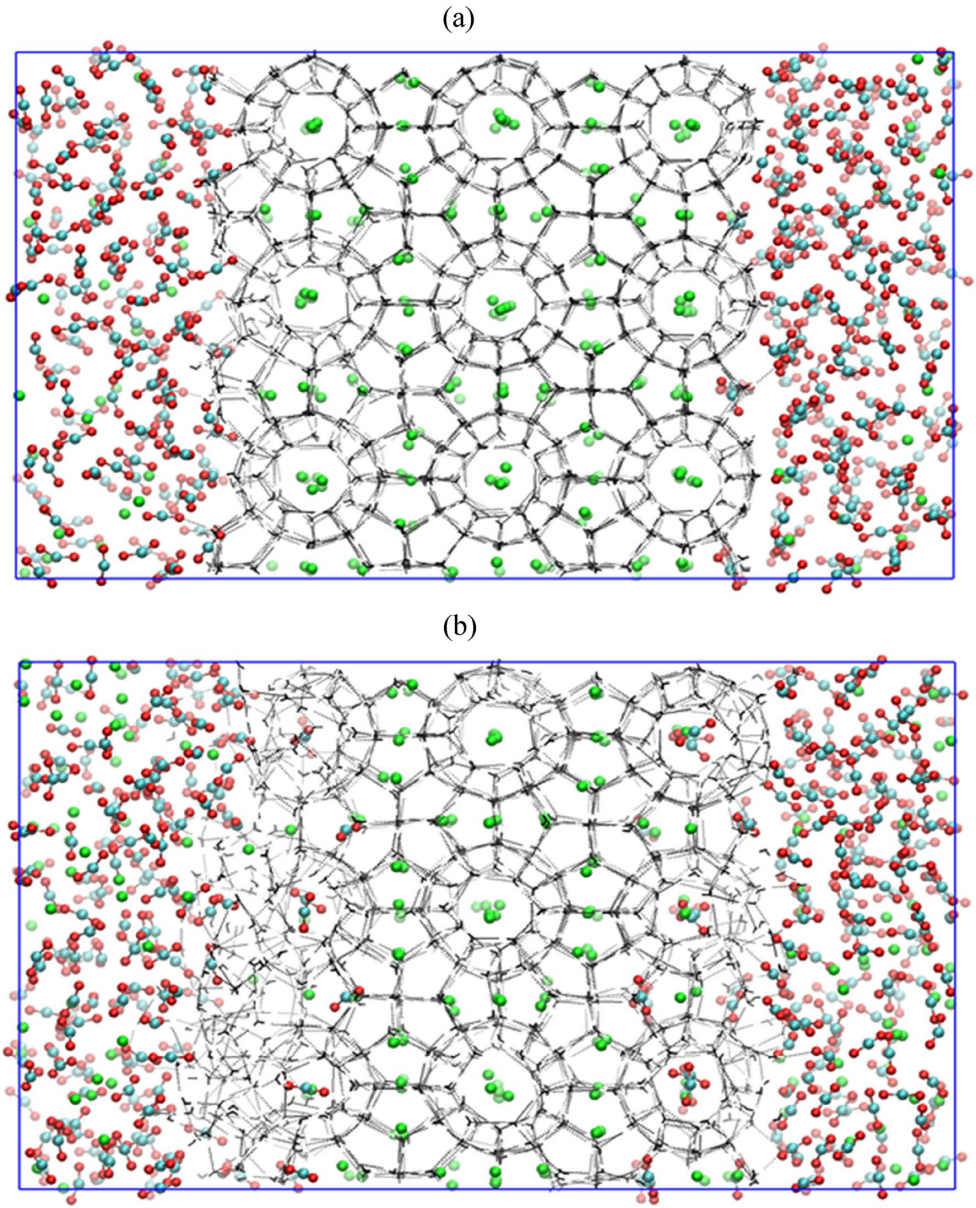
Screenshot of the CH_4_–CO_2_ replacement dynamics in absence of free water molecules with operating pressure and temperature of 50 MPa and 260 K at (**a**) 0 µs and (**b**) 2 µs.

To investigate the different mechanisms involved in the CH_4_–CO_2_ replacement, we perform the molecular dynamics simulation by using CO_2_ as a replacing agent in (i) absence of free water molecules, (ii) presence of free water molecules, and (iii) presence of free water molecules and salt ions. In the first and second configurations, we introduce the CO_2_ and water molecules on a random basis to fill out the available gap on the extended side of the 3 × 3 × 3 methane hydrate crystal. While in the third configuration, we introduce 3.5 wt% NaCl, a concentration matching typical seawater, in the aqueous CO_2_ solution. Figures 1a, 4a and 6a present the energy minimized initial configurations of these three operation modes. Note that every system is first brought to the desired temperature of 260 K and pressure of 50 MPa by conducting the NVT and NPT simulations for 1 ns and 5 ns, respectively, with position restraints imposed on the water molecules. Thereafter, the aforementioned systems are simulated for 2 µs of production run by removing the restraints on the water molecules. In the following subsections, we systematically present the CH_4_–CO_2_ replacement phenomena in the three above-mentioned techniques.

### Replacement of CH_4_ by CO_2_ in absence of free water molecules

Figure 1a depicts the state of the system after the equilibration of a simulation box of size 60 × 36 × 36 Å^3^, which comprises of a 36 × 36 × 36 Å^3^ supercell of methane hydrate and rest the CO_2_ molecules. Here, one can observe that the free methane molecules present at the face of the supercell are dispersed into the CO_2_ phase after minimizing the energy of the concerned system. However, this supercell marginally deviates from its original structure. This equilibrated configuration is then further utilized for the NPT production run of 2 µs. Figure 1b depicts the final state of the system at the completion of dynamic simulation in the absence of free water molecules at temperature of 260 K and pressure of 50 MPa. This figure shows that the methane occupying the surface cavities of the seed crystal is replaced with the CO_2_ molecules. Therefore, one can confirm that the present operating environment offers facilitation for CO_2_ hydrate growth than the reformation of CH_4_ hydrate. Besides, the replacement mechanism of CH_4_ with CO_2_ requires a careful analysis of the equilibrium bulk phase. The equilibrated system configuration provided in Fig. 1a confers a surface of maximum contact for the bulk CO_2_ with the seed methane hydrates. This hydrate-gas interface serves as a plane of maximum driving force for the molecular transfer. Consequently, from Fig. 1b, it is perceived that at the interface of CH_4_ hydrate supercell, CH_4_ is almost replaced by CO_2_ molecules at the end of 2 μs simulation. With the progress of CH_4_–CO_2_ replacement dynamics, it is observed that the water molecules of the host cavity bonded by H-bonds partially or completely detach. This hydrate lattice restructuring at the interface allows the CO_2_ molecule to enter and stabilize the hydrate cavities.

**Figure 2:**
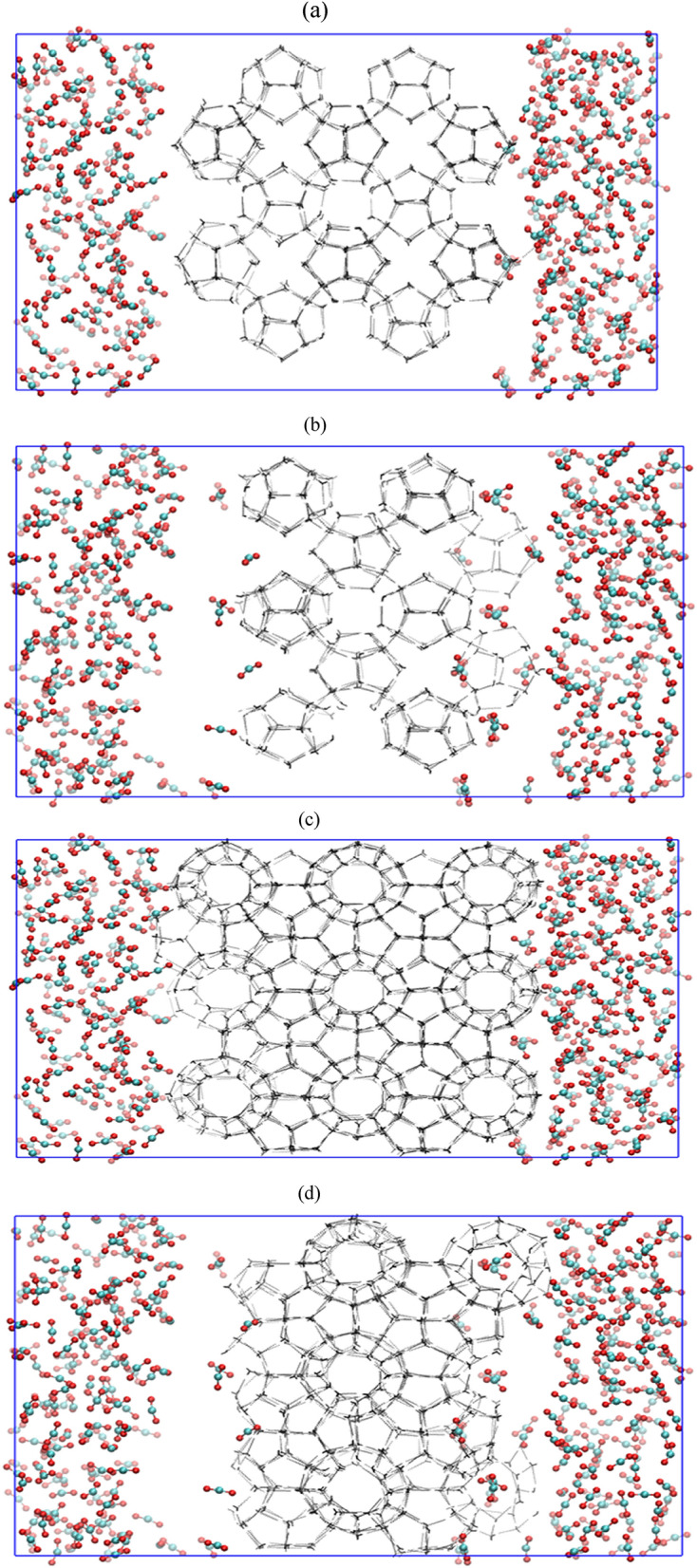
Cage-wise screenshot for the replacement of CH_4_ by CO_2_ in absence of free water molecules with operating pressure and temperature of 50 MPa and 260 K at (**a**) 0 µs (small cages), (**b**) 2 µs (small cages), (**c**) 0 µs (large cages) and (**d**) 2 µs (large cages).

Common occurrence of S-I type structure for pure hydrates of methane or carbon dioxide makes the occupied cages indistinguishable for CH_4_–CO_2_ replacement. Thus, one cannot visualize and quantify the distribution of CO_2_ occupancies in small and large cavities. To deal with this issue, we visualize the hydrate cavities independently and present this cage-wise distribution in Fig. 2a,b for small cages and, Fig. 2c,d for large cages at 0 and 2 μs, respectively. On comparing Fig. 2b,d, we observe that carbon dioxide molecules have a preference towards occupying the large cages than the small cages. This is attributed to the ratio of CO_2_ molecular size to cavity diameter of the large cages. Here, the ratios of guest to cavity diameter for small and large cages are 1.00 and 0.834, respectively^25^. Since, the size of small cavities equates the molecular size of CO_2_, these molecule experience severe steric hindrance towards its occupancy and thus, fails to stabilize the smaller cages. On the other hand, in case of large cages, there is a comparable difference between the size of CO_2_ molecule and cavity. Therefore, the CO_2_ molecule experiences less resistance to occupy and stabilize the large cavity of the hydrates.

**Figure 3:**
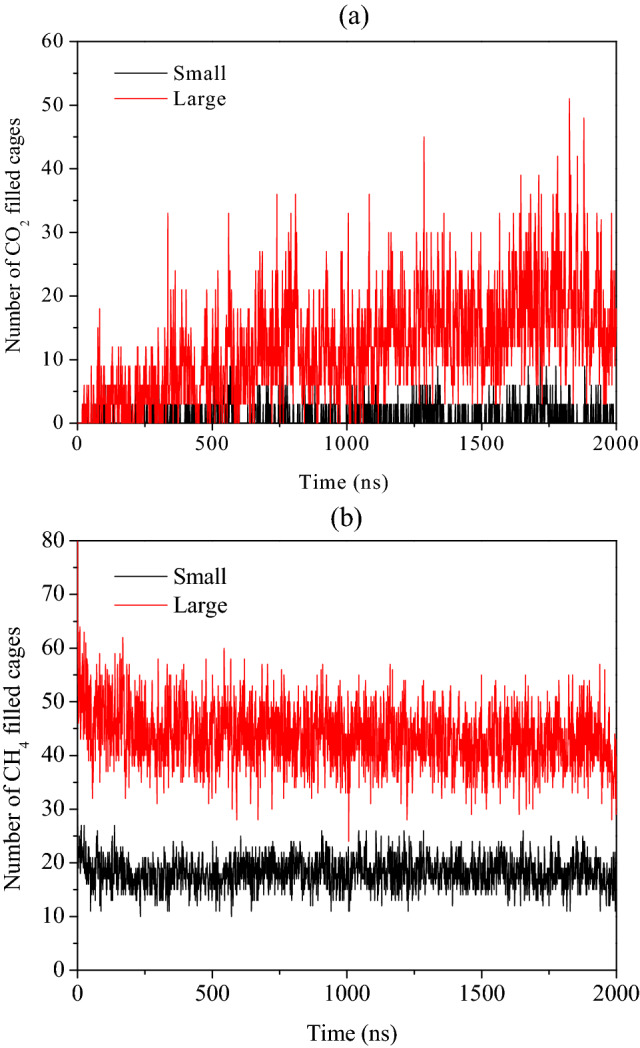
Plot of number of filled cages of (**a**) CO_2_ and (**b**) CH_4_ hydrates during the CH_4_–CO_2_ replacement dynamics in absence of free water molecules with operating pressure and temperature of 50 MPa and 260 K, respectively.

Now, to quantify the dynamic behavior of CH_4_–CO_2_ replacement, we provide the time evolution plots of the number of small and large cages occupying CO_2_ and CH_4_ molecules in Fig. 3a,b, respectively. From Fig. 3a, we observe that the number of CO_2_ molecules in the large cages increases with respect to replacement reaction run time. While for the small ones, the number of filled cages increases occasionally, followed by its return to the constant value. This also confirms the preferential nature of CO_2_ molecules to occupy the large cages of S-I hydrate structure. From Fig. 3b, one can observe a slight decrease in the number of small and large cages of CH_4_ hydrates for initial 20 ns run time. Thereafter, we notice fluctuating trends in the occupation of both cavity types of the hydrate lattice by the CH_4_ molecules. However, the higher magnitude of decrease in the number of large cages occupied by CH_4_ is evident in the initial phase of replacement reaction. This clearly signifies the existence of a direct replacement of CH_4_ by CO_2_ in absence of free water molecules. This mechanism is characterized by lack of complete breakage or dissociation of the hydrate cavities. Furthermore, we witness that the partial breakage of a cavity is enough to extract methane from the cavities and sequestrate carbon dioxide into the same.

### Replacement of CH_4_ by CO_2_ in presence of free water molecules

**Figure 4:**
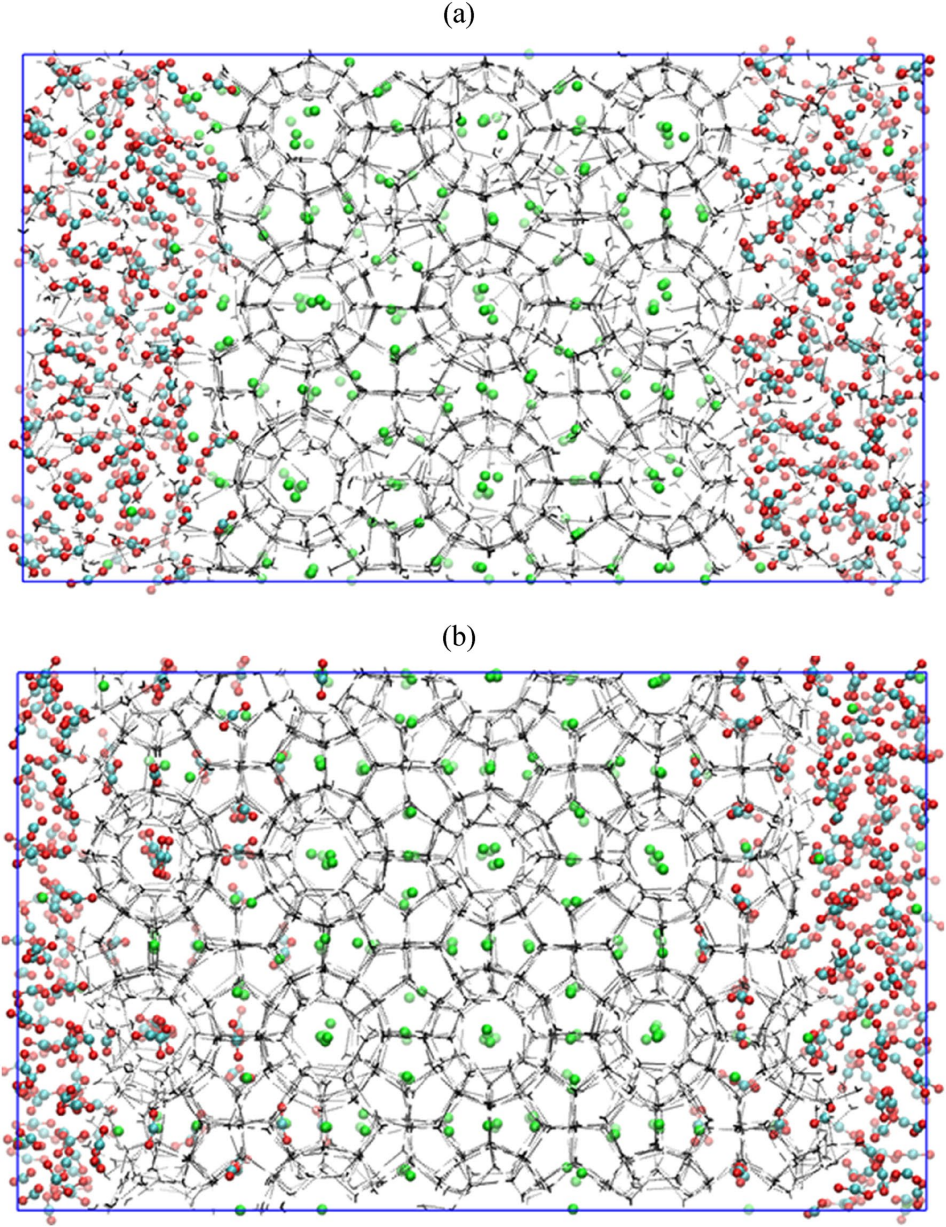
Screenshot of the CH_4_–CO_2_ replacement dynamics in presence of free water molecules with operating temperature and pressure of 260 K and 50 MPa at (**a**) 0 µs and (**b**) 2 µs. The water molecules are defined by TIP4P-Ew model.

Here, we investigate the effect of free water molecules on CH_4_–CO_2_ replacement dynamics at temperature and pressure of 260 K and 50 MPa, respectively. Figure 4a depicts the equilibrated initial configuration for CH_4_–CO_2_ replacement in presence of free water. Figure 4b provides a final state of this system at the end of 2 μs production run. The comparison between Fig. 4a,b suggests the predominant formation of new CO_2_ hydrate layers on account of its higher lattice stability at the concerned operating condition. These hydrate layers are observed to be built on the foundation of the mixed CH_4_–CO_2_ hydrates. Interestingly, the methane molecules in the vicinity of these newly formed cages are observed to be displaced by CO_2_ molecules. Moreover, these replaced methane molecules are observed to gradually transfer from the hydrate lattice towards CH_4_–CO_2_ mixed gas phase. Thus, with enough passage of time, CO_2_ dissolved in water shows the tendency to replace CH_4_ occupied in the hydrate cages. But unlike the previous case, as in the absence of free water molecules, CO_2_ molecules are observed to replace CH_4_ molecules by occupying both the large and small cages. However, in this configuration, the lattice distortion and breakage are fairly limited and a much stable CH_4_–CO_2_ mixed hydrate structure is observed. Naturally, the replacement magnitude is considerably lower as compared to the previous case. This can also be attributed to the fact that the present system has plenty of free water molecules that exhibit a higher affinity towards a hydrate structure with CO_2_. Therefore, carbon dioxide dissolved in the aqueous phase cannot significantly disturb the already existing methane hydrates. Thus, a predominant formation of CH_4_–CO_2_ mixed hydrates is observed in presence of free water. These free water molecules facilitate the growth of new CO_2_ hydrate layers without disrupting the stability of existing CH_4_ hydrates.

**Figure 5:**
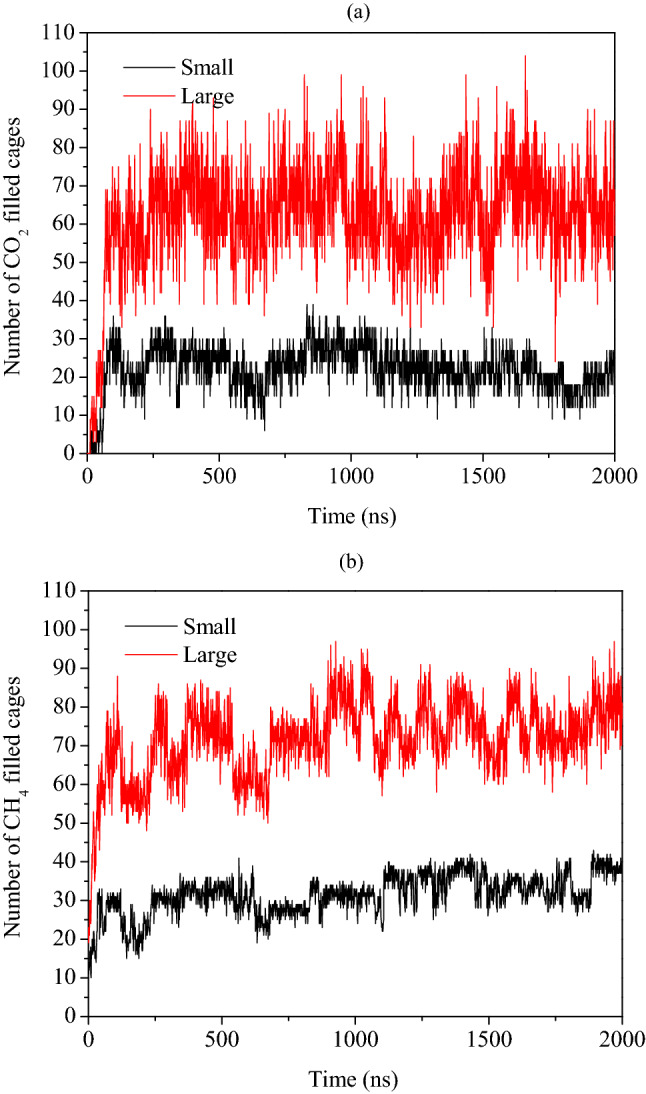
Plot of number of filled cages of (**a**) CO_2_ and (**b**) CH_4_ hydrates during the CH_4_–CO_2_ replacement dynamics in presence of free water molecules with operating pressure and temperature of 50 MPa and 260 K, respectively.

The number of cages occupied by CO_2_ and CH_4_ guests with the progress of replacement reaction is quantified in Fig. 5a,b, respectively. In Fig. 5a, we observe a small yet appreciable growth in the number of small cages occupied by CO_2_ molecules; whereas, a considerable increase in the number of large cages occupying CO_2_ guests is observed. These both observations can be attributed to the united effect of the formation of new layers of mixed hydrates and the replacement of CH_4_ by CO_2_ in the existing hydrates.

By studying CH_4_ occupancies in small and large cages of the hydrate structure, in Fig. 5b, we observe incremental behavior for the initial period of replacement reaction. However, after a threshold these occupancies stagnate and can be attributed to the reformation of hydrate lattices by the displaced CH_4_ molecules. Unlike the previous case, here, we notice an increment in the number of occupied cages both small and large by CH_4_ molecules. Therefore, one can put forward that the presence of free water molecules has facilitated the reformation of CH_4_ hydrates, which can certainly affect the overall CH_4_–CO_2_ replacement efficiency.

### Replacement of CH_4_ by CO_2_ in presence of free water molecules and salt

**Figure 6:**
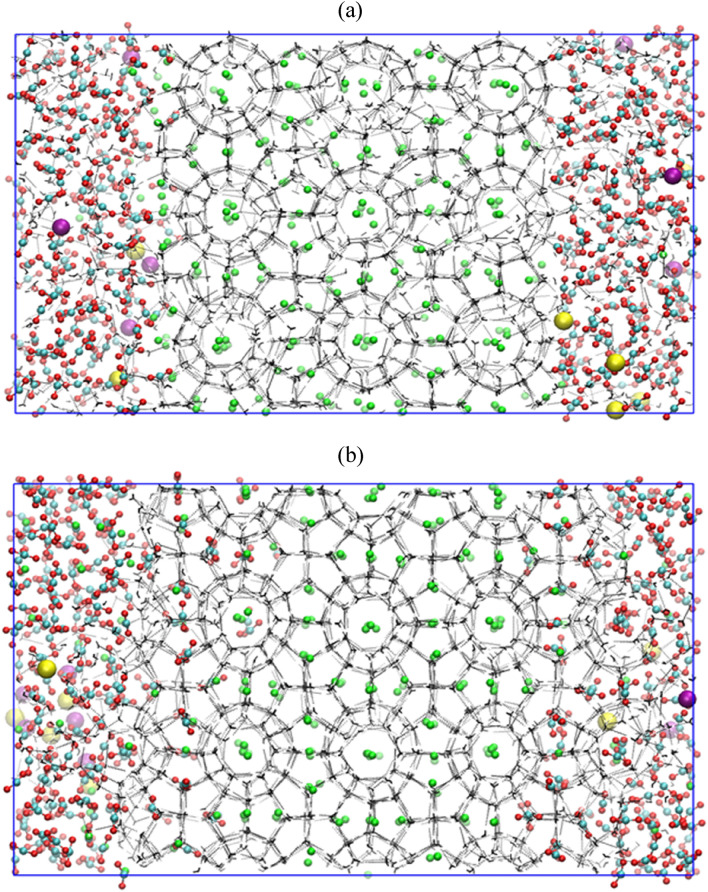
Screenshot of the CH_4_–CO_2_ replacement dynamics in presence of free water molecules, carbon dioxide and salt ions with operating conditions of 260 K and 50 MPa at (**a**) 0 µs and (**b**) 2 µs. The atoms of sodium and chlorine ions are represented by purple and yellow spheres, respectively. The water molecules are defined by TIP4P-Ew model.

**Figure 7:**
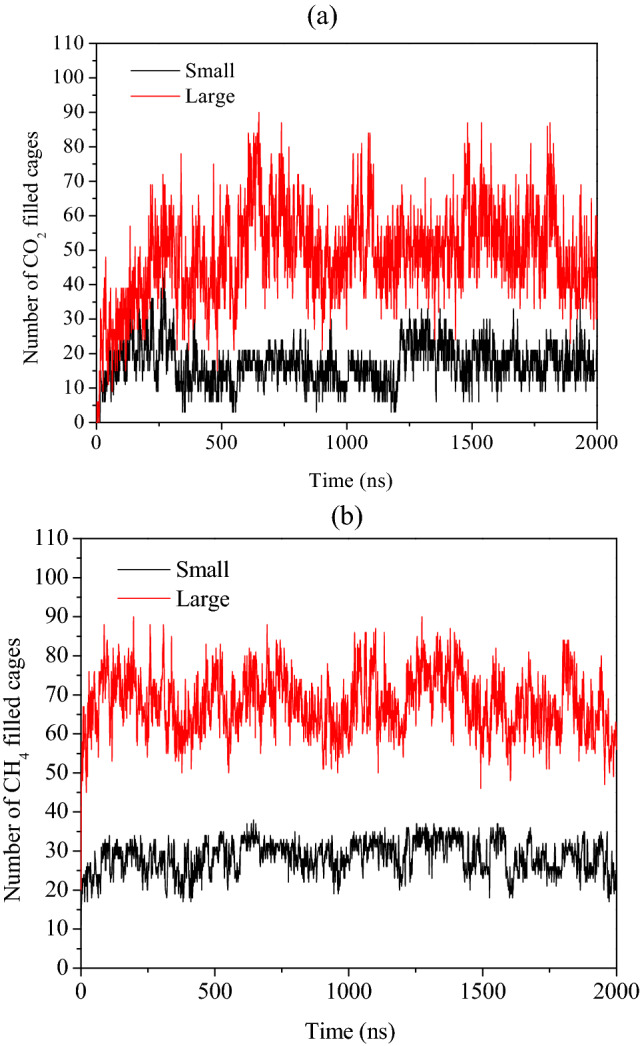
Plot of number of filled cages of (**a**) CO_2_ and (**b**) CH_4_ hydrates during the CH_4_–CO_2_ replacement dynamics in presence of free water molecules, carbon dioxide and salt ions with operating pressure and temperature of 50 MPa and 260 K, respectively.

Here, we evaluate the combined influence of water, replacing agent CO_2_ and salt on CH_4_–CO_2_ replacement dynamics. For this, we use a 3.5 wt% aqueous NaCl solution for representing the typical saline water systems. Figure 6a represents a system configuration after 1 ns NVT and 5 ns NPT equilibration runs, which is utilized for further production run of CH_4_–CO_2_ replacement at temperature and pressure of 260 K and 50 MPa, respectively. Figure 6a illustrates that the presence of salt ions in the system already distorts the host lattice, paving way for the replacement of methane with CO_2_. This also can be confirmed by comparing Figs. 1a, 4a and 6a, in which one can clearly visualize a slightly disturbed parent crystal of methane hydrates in presence of salt ions. Figure 6b provides a state of the concerned system after 2 µs CH_4_–CO_2_ replacement reaction. This figure illustrates the inhibitory nature of the salt towards the formation of mixed CH_4_–CO_2_ hydrates. The interesting fact about this case is that in spite of the presence of salt ions, the guest gases i.e. CO_2_ and CH_4_ have successfully formulated the hydrate structures. However, on comparing Figs. 4b and 6b, we notice that the CH_4_–CO_2_ mixed hydrate growth is limited in presence of salt ions as compared to the system of pure water and CO_2_. This is attributed to the inhibiting behavior of salt ions towards the hydrate growth. Now, to quantify this effect towards CH_4_–CO_2_ replacement dynamics, Fig. 7a,b presents the progress of the formation of a number of small and large cages of CO_2_ and CH_4_ hydrates. In case of Fig. 7a, initially, the small cages of CO_2_ hydrates show a peak indicating the unstable nature of such cages, which further falls to certain extent and thereafter it remains stable with respect to time. However, the number of large cages is observed to significantly grow with time, which also stabilizes after 600 ns. Concurrently from Fig. 7b, we observe that the presence of salt ions has also significantly lowered the growth of small and large cages of CH_4_ hydrate. From the aforementioned observations, it can be perceived that the salt ions have significantly affected and lowered the formation of CH_4_–CO_2_ mixed hydrates, and consequently, the overall replacement mechanism.

**Figure 8:**
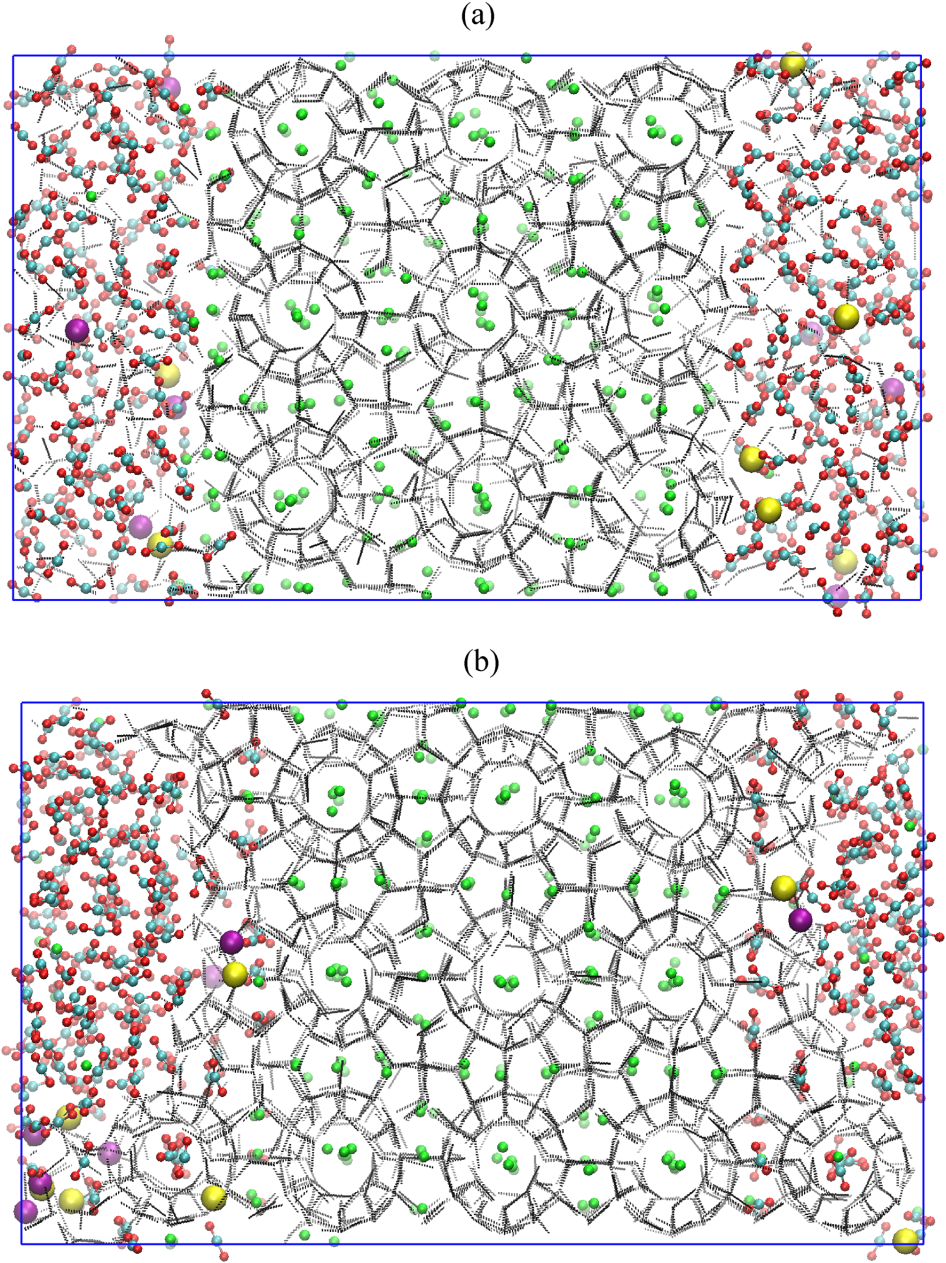
Screenshot of the CH_4_–CO_2_ replacement dynamics in presence of free water molecules, carbon dioxide and salt ions with operating conditions of 260 K and 50 MPa at (**a**) 0 µs and (**b**) 2 µs. The atoms of sodium and chlorine ions are represented by purple and yellow spheres, respectively. The water molecules are defined by TIP4P-Ice model.

**Figure 9:**
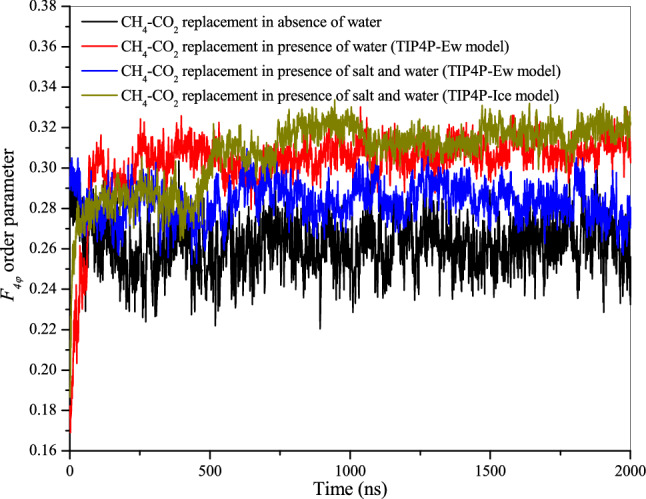
*F*_*4φ*_ order parameter for the CH_4_–CO_2_ replacement in absence and presence of water and salt ions at 260 K and 50 MPa.

For the aforementioned simulation experiments, we have used Ewald modified transferable intermolecular potential with 4 points to define the water molecules. However, it is essential to evaluate the influence of another water model which can probably account the ice phase diagram. Therefore, we have used TIP4P-Ice model to define the water molecules. Figure 8 presents the screenshots of the simulation results of CH_4_–CO_2_ replacement dynamics at the start (0 µs) and end (2 µs) of experiment. Note that, the operating conditions employed in this experiment are identical with previous case. For gaining further insight into the structural progress of methane-carbon dioxide replacement process, we evaluate and compare the order parameter of the concerned simulation experiments. In this context, Fig. 9 illustrates the *F*_*4φ*_ order parameter plots for the CH_4_–CO_2_ replacement process in absence and presence of water and salt ions. Here, we clearly observed the differences in the structural development. Overall, the presence of free water molecules have resulted an ordered growth of CH_4_–CO_2_ mixed hydrate.

**Figure 10:**
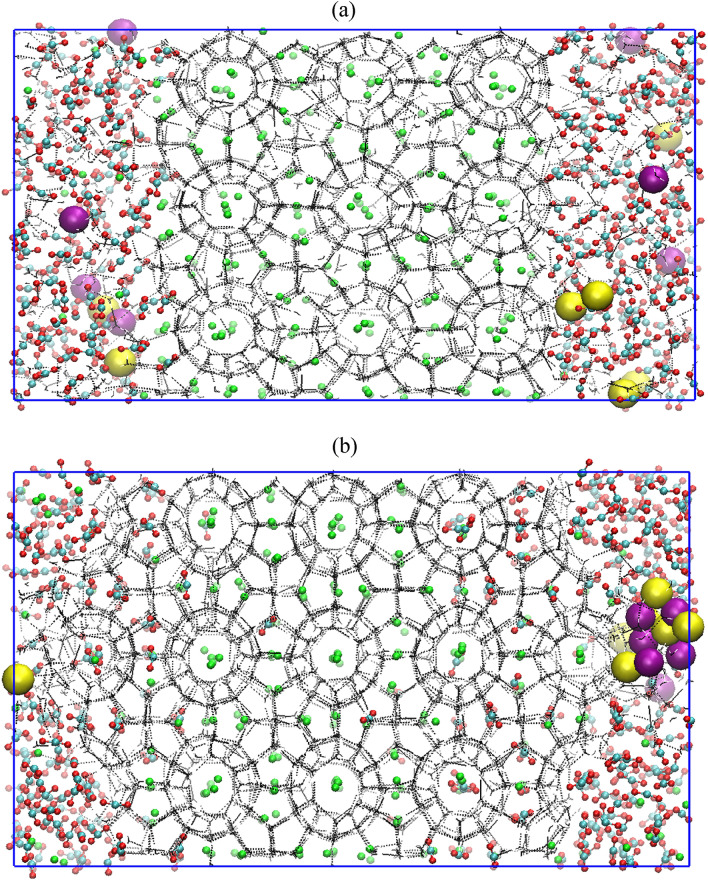
Screenshot of the CH_4_–CO_2_ replacement dynamics in presence of free water molecules, carbon dioxide and salt ions with operating conditions of 260 K and 10 MPa at (**a**) 0 µs and (**b**) 2 µs. The atoms of sodium and chlorine ions are represented by purple and yellow spheres, respectively.

**Figure 11:**
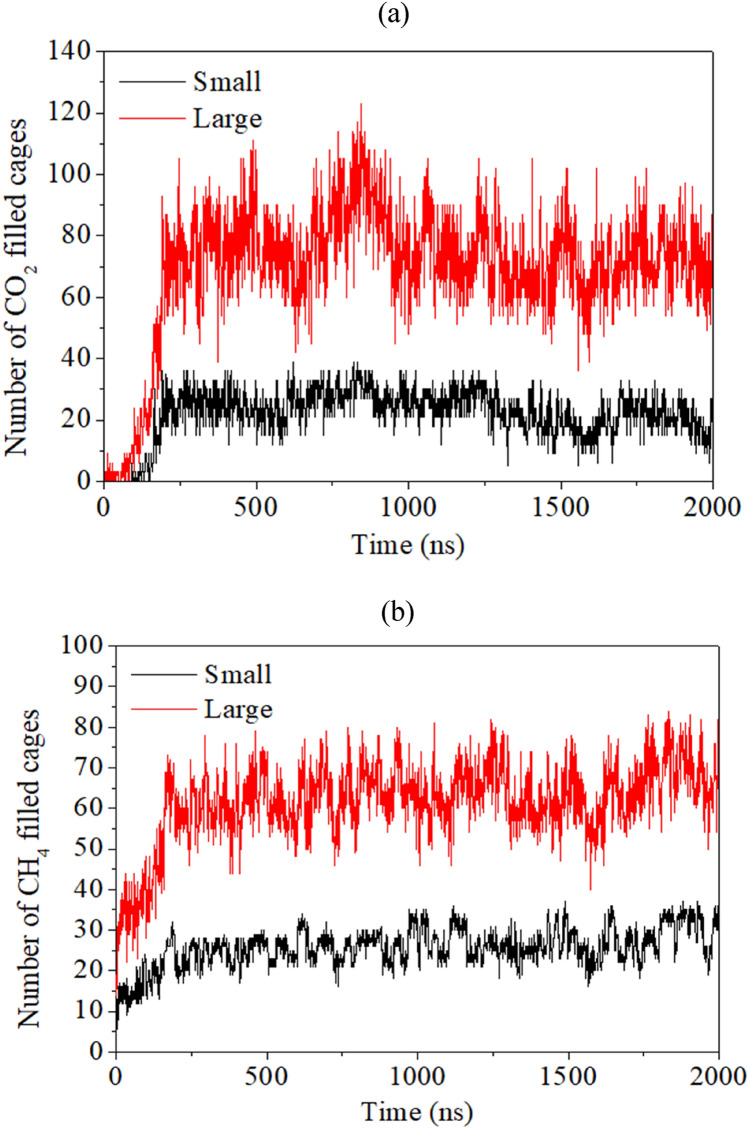
Plot of number of filled cages of (**a**) CO_2_ and (**b**) CH_4_ hydrates during the CH_4_–CO_2_ replacement dynamics in presence of free water molecules, carbon dioxide and salt ions at operating temperature and pressure of 260 K and 10 MPa, respectively.

Finally, in the view of examining the CH_4_–CO_2_ replacement phenomenon in the realistic environment, we evaluate the effect of replacing agent—CO_2_, free water and salt ions by lowering the operating pressure to 10 MPa, while keeping the same temperature (260 K) as in the previous cases. The screenshots of the present system at initial (0 µs) and final (2 µs) time of NPT production run are provided in Fig. 10. Examining these snapshots, one clearly observes that CO_2_ molecules have considerably replaced CH_4_ molecules. Interestingly, the CO_2_ molecules are able to penetrate the first layer of methane hydrate. Here, two factors are favoring the formation of CO_2_ hydrate over CH_4_ hydrate: firstly, the presence of salts and secondly, the low operating pressure. Furthermore, Figs. 11a,b quantify the aforementioned observations in terms of the number of CH_4_ and CO_2_ filled hydrate cages. Compared to the previous case, comprising of 50 MPa operating pressure, an increased number of CO_2_ hydrate cages are found in the present case (10 MPa). This observation supports the fact that the reduction in operating pressure enhances CO_2_ hydrate formation over the reformation of CH_4_ hydrate and thereby, it can contribute to enhance the overall replacement mechanism.

## Concluding remarks

In this contribution, we investigate the CH_4_–CO_2_ replacement mechanism in three modes i.e., CO_2_ as a replacing agent in (i) absence of free water molecules, (ii) presence of free water molecules, and (iii) presence of saline water solution at temperature and pressure of 260 K and 50 MPa, respectively. In the first mode of replacement mechanism, carbon dioxide is observed to distort the first layer of the existing methane hydrate crystals and replace the methane in those cavities. While, in the second and third modes, we observe more number of CO_2_ hydrate formation with CH_4_ replaced in those cavities. However, the number of cavities filled by methane molecules also shows an initial rise with time due to their reformation tendency. The presence of salt favors the destabilization of methane hydrate cages that eventually allows the reformation of the CO_2_ filled cavities. Finally, in the view of representing the reservoir scale CH_4_–CO_2_ replacement mechanism, we perform a microsecond level dynamic simulation at a pressure of 10 MPa in presence of CO_2_ and 3.5 wt% saline water solution. Here, we noticed a significant facilitation of CO_2_ hydrate growth than the reformation of CH_4_ hydrates, which consequently favored the objective of the replacement mechanism. In this way, the present findings can useful in understanding the key mechanisms involved in an ecofriendly recovery of natural gas from its hydrate reservoirs.

## Methods

### Computational details

#### System formation

The basic configuration of the simulation box is constructed from a perfect methane hydrate lattice. We adopt the coordinates of water and methane molecules, representing the S-I hydrate structure, from Takeuchi et al.^26^. The unit cell has dimensions of 12 × 12 × 12 Å^3^ and features guest molecules (methane) residing at the centers of each cavity. A 3 × 3 × 3 supercell is built from the unit cell and is then expanded on either side along the *x*-direction by 12 Å. The resultant or obtained 60 × 36 × 36 Å^3^ volume serves as the basic configuration for this molecular dynamic study. The expanded free volume is filled with (i) pure carbon dioxide, (ii) carbon dioxide + water, and (iii) carbon dioxide + water + 3.5 wt% NaCl to investigate diverse replacement mechanisms. The molecular dynamics simulations are carried out for each of the formulated configurations using the single precision GROMACS software, version 2018.1^27^.

After these configurations are generated, the molecules are modeled using appropriate forcefield models. In this view, the water molecules are defined with the TIP4P-Ew model, which stands for the ‘Ewald modified transferable intermolecular potential with 4 points’^28^. Besides, the TIP4P-Ice model is also employed for water molecules. The water molecules are kept rigid using the algorithm of linear constraint solver (LINCS). The rest of the molecules are defined with the ‘optimized potentials for liquid simulations’ (OPLS) model^29^. Among these molecules, the united atom (OPLS-UA) forcefield is used for methane molecules, while carbon dioxide and NaCl molecules are defined with their all-atom (OPLS-AA) variant. In GROMACS, the interaction between any two sites is defined with the pair additive form of the Lennard–Jones potential model as:

1 $$ E(r_{ij} ) = \sum\limits_{i} {\sum\limits_{i} {\left\{ {4\varepsilon_{ij} \left[ {\left( {\frac{{\sigma_{ij} }}{{r_{ij} }}} \right)^{12} - \left( {\frac{{\sigma_{ij} }}{{r_{ij} }}} \right)^{6} } \right] + \left( {\frac{{q_{i} q_{j} }}{{r_{ij} }}} \right)} \right\}} } $$

The model possesses the parameters that are collision diameter (*σ*), energy well depth (*ε*) and Coulombic charge (*q*), while the summations over the *i*, *j* sites compute the cumulative potential. The interaction parameters for the salt (sodium chloride) are presented in Table 1.

**Table 1:** Forcefield parameters for sodium chloride^29^.

Molecule	Atom type	Mass	*σ* (Å)	*ε* (kJ mol^−1^ )	*q* (e)
Sodium chloride	Na	22.990	1.897440	6.724270	+ 1.000000
	Cl	35.453	4.417240	0.492833	− 1.000000

#### Molecular dynamics simulation

Molecular dynamics is based on Newton's law of motion, where the displacement of a particular atom is quantified by the resultant force that the atom is experiencing due to its interaction with the surrounding atoms. For this purpose, the systems formed in the previous subsection are required to gain the minimum energy configuration. The algorithm for energy minimization utilizes the steepest descent method. The periodic boundary condition is applied in all three dimensions to ensure mass conservation in the system. The cutoff radius for the short-range electrostatic potential and the van der Waals interactions are defined to be 10 Å, while the particle mesh Ewald (PME) method is applied for the long-range electrostatic potential.

After the system is brought to the minimized state, the first molecular dynamics simulation is run in NVT (constant numbers of components, temperature and volume) ensemble for 1 ns to bring the system to the desired operating temperature. For this purpose, the Nose–Hoover thermostat is utilized with a 0.1 ps time constant. The position restrained is imposed on the system species so that the atoms do not move from their respective positions in this equilibration step. Thereafter, another equilibration is performed in NPT (constant numbers of components, pressure and temperature) ensemble for 5 ns to bring the system to the desired operating pressure. In this step, Parrinello–Rahman barostat with a 0.5 ps time constant is utilized along with the previous setting of the thermostat. It may be noted that the system is kept position restrained in this step as well for the same reason stated in the previous equilibration step. Eventually, the production run is performed in the NPT ensemble by removing this position restraint. For the accuracy in dynamics of the system, the step sizes for all these equilibration and production runs are set as 1 fs. To allow the carbon dioxide to replace the methane from the hydrate network, this simulation is performed for 2 µs.

#### Post simulation analysis

In the production run, the frames at each nanosecond are recorded for post-simulation analysis. This includes both the visualization and quantification of methane and carbon dioxide filled cavities. The visualization is performed in VMD software, while the quantification is performed using the GRADE^30^ software. GRADE distinguishes between the 5^12^, 5^12^6^2^ and 5^12^6^4^ cages types based on the number of water molecules and, hexagonal and pentagonal rings.

## Data Availability

The data that support the findings of this study are available from the corresponding author on reasonable request.
